# Matched Paired Primary and Recurrent Meningiomas Points to Cell-Death Program Contributions to Genomic and Epigenomic Instability along Tumor Progression

**DOI:** 10.3390/cancers14164008

**Published:** 2022-08-19

**Authors:** Teresa San-Miguel, Javier Megías, Daniel Monleón, Lara Navarro, Lisandra Muñoz-Hidalgo, Carmina Montoliu, Marina Meri, Pedro Roldán, Miguel Cerdá-Nicolás, Concha López-Ginés

**Affiliations:** 1 Department of Pathology, Medical School, INCLIVA, University of Valencia, 46010 Valencia, Spain; 2 Department of Pathology, Hospital General Universitario, 46014 Valencia, Spain; 3 Department of Neurosurgery, Hospital Clínico Universitario, 46010 Valencia, Spain

**Keywords:** meningioma, recurrence, *HIC1*, *CDKN2B*, *DAPK1*, *GSTP1*

## Abstract

**Simple Summary:**

Meningioma (MN) is the most frequent primary brain tumor with a high frequency of recurrences and a lack of objective tools for predicting their prognosis. In this study, we analyzed a careful selection of patients in which both the primary tumor and at least one recurrence were available, allowing us to extend the changes that occur during tumor progression. We developed a histological, genetic, and epigenetic analysis of the samples. Thus, we identified markers of quick recurrence, increased tumor instability by copy number alterations, and the accumulation of epigenetic changes during tumor progression. Interestingly, the genes involved seemed to be randomly distributed along the genome but eventually suggest a common impact on cell-death programs such as apoptosis and autophagy.

**Abstract:**

Meningioma (MN) is an important cause of disability, and predictive tools for estimating the risk of recurrence are still scarce. The need for objective and cost-effective techniques addressed to this purpose is well known. In this study, we present methylation-specific multiplex ligation-dependent probe amplification (MS-MLPA) as a friendly method for deepening the understanding of the mechanisms underlying meningioma progression. A large follow-up allowed us to obtain 50 samples, which included the primary tumor of 20 patients in which half of them are suffering one recurrence and the other half are suffering more than one. We histologically characterized the samples and performed MS-MLPA assays validated by FISH to assess their copy number alterations (CNA) and epigenetic status. Interestingly, we determined the increase in tumor instability with higher values of CNA during the progression accompanied by an increase in epigenetic damage. We also found a loss of *HIC1* and the hypermethylation of *CDKN2B* and *PTEN* as independent prognostic markers. Comparison between grade 1 and higher primary MN’s self-evolution pointed to a central role of *GSTP1* in the first stages of the disease. Finally, a high rate of alterations in genes that are related to apoptosis and autophagy, such as *DAPK1*, *PARK2*, *BCL2*, *FHIT*, or *VHL*, underlines an important influence on cell-death programs through different pathways.

## 1. Introduction

Meningiomas (MN) are the most frequent primary intracranial tumors [1,2]. They cause many surgical procedures every year, and despite being usually benign, their delicate location makes them a lurid cause of disability. It is disconcerting that after initial successful treatments, the overall recurrence rate is above 20% of the cases [3,4]. In other words, after the first neurosurgery, many patients are condemned to a second one with all clinical consequences affecting their wellness. The diagnosis of meningioma relies on morphological features related to their tendency to recur. The last edition of the WHO’s classification describes 15 different meningioma subtypes: 9 grade 1 MN that show slow growth rates and benign biological behavior, 3 grade 2 MN, which show an increased risk of recurrence, and 3 grade 3 MN, displaying an aggressive clinical outcome and elevated recurrence rates. In addition, some molecular biomarkers such as *TERT* promoter mutation and/or the homozygous deletion of *CDKN2A/B* have now been included as the criteria for the diagnosis of grade 3 MN because of their associations with tumor aggressiveness [1].

The loss of chromosome 22 and/or del (22q) is by far the most frequent chromosomal alteration in meningiomas and involves the *NF2* tumor-suppressor gene (TSG). Its alteration is an early event in all WHO grades, and it is relevant for MN development and progression [5]. Higher-grade MN usually exhibit more complex genetic changes, with losses on 1p, 6p/q, 10q, 14q, and 18p/q, and the deletions of *CDKN2A/B*, in which the latter is confirmed as a progression event in *NF2*-altered mice models [1,6]. Genomic sequencing of different series of sporadic MN defined two subsets of MN according to *NF2* status: Mutated or lost *NF2* characterizes the first while different alterations in *AKT1* or *SMO*, *TRAF7*, *KLF4*, or *PIK3CA* characterizes the second. In non-*NF2* MN, single mutations of *TRAF7*, *AKT1*, *KLF4*, and *SMO* (TRAKLS mutation genotype) or combinations of some of them seem to be associated with WHO grade 1 and favorable progression-free survival [7,8,9,10,11]. Conversely, MNs with altered *NF2* are more likely to be atypical, and additional copy number alterations or general genomic instability tend to be more frequent in this MN group [7,8].

In this context, the genes recently involved in MN have been mainly linked to WHO grade 1 MN, displaying a favorable outcome. However, grade 1 MN causes the majority of recurrences in absolute numbers, emphasizing that there is still scarce knowledge on how to predict which grade 1 MN will behave in an aggressive manner [2]. An interesting approach for improving our understanding is the study of matched primary and recurrent samples, although there are not many large series previously reported. Recurrent meningiomas constitute a major health problem and the follow-up of the patients is problematic. A common situation in patients diagnosed as grade 1 MN is that, after the radiological identification of a recurrence, clinicians are cautious and ask the patients to wait in order to observe an evolution. This fact leads many patients to seek a second opinion to achieve surgical resections rapidly, complicating the generation of a large series of matched primary-recurrent MNs. In fact, we can find previous studies with many tumor samples collected over 10 years but the studies only have a small percentage of paired recurrences [9]. The present study addresses this problem and focuses on paired primary and recurrent tumors. We aim to retrospectively characterize primary tumors that have recurred and study their genetic and epigenetic landscape both on the primary neoplasm and in their recurrences. The strength of this study is the long period of sample collection that allow us to present a series of paired samples of meningioma from 20 patients. Our cohort includes primary tumors and one or more recurrences, reaching a total of 50 paired samples. In these samples, we offer a picture of copy number alterations (CNAs) and epigenetic aberrations (HMs) to better understand the characteristics of these recurrent meningiomas from both grade 1 and grade 2–3 primary neoplasms.

## 2. Materials and Methods

### 2.1. Patients, Samples, and Clinical Study

Tumor samples from 20 patients diagnosed with meningioma from the Hospital Clínico Universitario in Valencia (HCUV) were collected between 1986 and 2011. They comprised 50 samples: 20 primary tumors (PT) and the recurrences (RCs) suffered by these patients. First, RC was achieved from all patients except one (19 RC1). In addition, 6 matched second recurrences (RC2), 3 samples from third recurrences (RC3), and 2 fourth recurrences (RC4) were included in this study. Globally, 20 PTs and 30 RCs were collected (50 samples). For the case in which RC1 was not accessible, clinical data and the second recurrence were available. The study was conducted according to the guidelines of the Declaration of Helsinki and approved by the Institutional Ethics Committee at the University of Valencia and Hospital Clínico Universitario de Valencia (protocol 2014/183). Clinical information was accessed from the historical archive of the hospital, including data on age, sex, and recurrence-free survival period (RFS). None of the patients received chemotherapy or radiotherapy before the first surgery. After surgery, tumor specimens were fixed in neutral-buffered formalin, embedded in paraffin, sectioned, and stained with hematoxylin-eosin. Samples were categorized according to the WHO classification [1]. Among the histopathological features, mitoses were counted on 10 high-power fields (10 HPF), and the presence of prominent nucleoli, increased cellularity, necrosis, the infiltration of the dura-matter (or CNS adjacent structures), and sheeting was determined.

### 2.2. Immunohistochemistry

Immunostaining by the avidin-biotin-peroxidase method was carried out in order to evaluate the proliferation index through Ki-67 expression. It was evaluated by MIB-1 antibody staining (Agilent, Dako, Glostrup, Denmark) and was calculated by determining the percentage of immunopositive nuclei in two different areas. The results were categorized as 1 (<1% stained nuclei), 2 (1–5% stained nuclei), 3 (5–10% stained nuclei), and 4 (>10% stained nuclei), as previously described [12].

### 2.3. Molecular Analysis

Selected areas of the paraffin blocks from each sample were used for DNA extraction using the QIAamp DNA FFPE tissue kit (Qiagen, Inc., Valencia, CA, USA). The quality and quantity of DNA improved by using the standard ethanol precipitation procedure in all tumor and control samples. Multiplex ligation-dependent probe amplification using SALSA MLPA Probemix P044-NF2 kit (MRC-Holland, Amsterdam, The Netherlands) was performed following the manufacturer’s instruction to assess the genetic status of NF2. This kit included 17 probes for all the exons of the gene, and CNAs of NF2 were determined as its average value. A threshold of x < 0.75 was established to classify losses and 0.7 < x < 1.3 was considered wild-type (wt) based on previous descriptions [12]. Methylation-specific multiplex ligation-dependent probe amplification (MS-MLPA) was performed to determine the methylation status of 24 genes using a SALSA MLPA kit (ME001-C2, lot 0808), following the manufacturer’s instructions (MRC-Holland). The genes included were TP73*, *CASP8*, *FHIT*, RASSF1*, *VHL*, MLH1*, *CASR*, RARB*, *APC*, ESR1*, *PARK2*, CDKN2A*, CDKN2B*, DAPK1*, *CREM*, PTEN*, *CD44*, GSTP1*, *CD27*, ATM*, *PAH*, BRCA2*, *MLH3*, *TSC2*, CDH13*, HIC1*, *BCL2*, *KLK3*, and TIMP3*. The (*) points to the ones for which its probes allowed us to determine both the CNA and the methylation status (HM). They were mainly TSGs that were selected because they are already known to display frequently genetic alterations in meningioma but little is known about their epigenetic status or vice versa (e.g., *CDKN2A/B*, *PTEN*, *TIMP3*, *CD44*, *RASSF1*, and *TP73*). Other genes have important functions in different cancer-related processes, e.g., the regulation of tumor growth, cell-cycle control, differentiation and proliferation, angiogenesis, cell adhesion, DNA damage repair, and apoptosis [12,13,14,15,16]. Briefly, DNA was denatured at 98 °C for 5 min and hybridized with the appropriate probe mix at 95 °C for 1 min followed by a 60 °C overnight incubation. Ligation and digestion reactions with Hha I were carried out at 48 °C for 30 min followed by a step at 98 °C for 5 min. PCR was performed using the SALSA PCR primer mix and SALSA polymerase and consisted of 35 cycles of 95 °C/30 s, 60 °C/30 s, and 72 °C/1 min with a final step at 72 °C/20 min (all reagents from MRC-Holland). The thresholds established were x < 0.75 as losses, 0.7 < x < 1.3 as normal, 1.3 < x < 2 as unspecific, and x > 2 as gains, according to previous reports [12,15]. The studied genes that were wt in all the samples were removed from the data shown. The most frequent CNA detected was the loss of one allele; however, in order to refer to all alterations detected, including sporadically homozygous deletions or gains, we refer to them as CNA.

The amplified fragments were separated by capillary electrophoresis in an ABI 310 Sequencer (Applied Biosystems, Inc., Foster City, CA, USA) and were analyzed with Coffalyser excel-based software (MRC-Holland). Data were intra-normalized and results above 20% were considered positive for promoter hypermethylation, as previously described [12,16]. We used three non-related blood samples from healthy donors as negative controls. MLPA results were analyzed to obtain information about what genes were genetically or epigenetically affected in meningioma. Furthermore, based on previous reports, we took into account the total amount of genetic and epigenetic changes per case to determine the copy number’s alteration burden [15,17] and also the epigenetic burden.

### 2.4. Fluorescence In Situ Hybridization

Fluorescence in situ hybridization (FISH) studies for chromosomes 1, 14, and 22 were performed to validate the MLPA kits used. A random cohort of 50% of the paraffin-embedded samples was selected. Non-neoplastic tissues from the brain were used as control. To carry out the FISH analysis probes, LSI 22q12, LSI 1p36/LSI 1q25, and t (11;14) IGH/CCND1 were used according to the manufacturer’s instructions (Vysis, Abbot scientific, Madrid, Spain). The process of counterstaining nuclei was performed using DAPI. The fluorescent signals were detected using a Leica LAS AF photomicroscope with appropriate filters. Signals were counted in a range of 100–150 non-overlapping tumor cell nuclei per case. An interpretation of deletion was made when >20% of the nuclei harbored losses based on the cutoffs established in control samples [18]. FISH probe LSI 22q12 was compared to the data obtained from the P044-NF2 kit. The FISH analysis of chromosomes 1 and 14 was compared to the values obtained from the *TP73* and *MLH3* MLPA probes from the ME001-C2 kit, respectively, which are located on 1p36 and 14q24.3, respectively. Cohen’s Kappa (K) statistic was used to determine the agreement between both assessments. 0 < K < 0.2 was considered as slight agreement, 0.2 < K < 0.4, as fair agreement, 0.41 < K < 0.60 as moderate agreement, 0.61 < K < 0.8 as substantial agreement, and 0.81 < K < 1 as almost perfect agreement.

### 2.5. Statistics

Statistical analysis was performed with IBM SPSS v. 24 software (IBM, Madrid, Spain). When possible, the variables were categorized. Quantitative variables were evaluated by Kolmogorov–Smirnov and Levene tests; depending on their results, Student’s T, Mann–Whitney’s U, or Kruskal–Wallis tests were carried out. Categorical variables were evaluated using the Chi-square (χ^2^), Fisher’s exact, and Cramer’s V statistic tests depending on their characteristics. Bivariate correlation analysis was performed using Pearson’s statistic for association among variables. Significance was accepted when the probability level was *p* < 0.050. Kaplan–Meier curves were built in SPSS for primary tumors stratified by genetic alterations to evaluate differences in recurrence-free survival. In addition, we built Cox regression hazards models to evaluate these genetic alterations as independent prognostic factors in primary meningiomas.

## 3. Results

### 3.1. Clinical Data and Histopathological Results

This study analyzed 50 samples that came from 20 patients: 60% were men and 40% were women. Patient age at diagnosis ranged from 7 to 68 years, with an average of 52.0 ± 3.4 years. Of note is that 70% of the cases were under 60 years old at diagnosis. The primary tumors were located at the sphenoid wing in 20% of the patients; in the olfactory groove and frontal and parietal location in 15% of patients; in posterior cranial fossa, parasagittal, and occipital in 10% of patients; and ventricular location in 5% of patients. The average tumor size was 5.3 cm^3^, ranging from 2 to 8 cm^3^. Upon initial diagnoses, the standard treatment consisted of maximal surgical resection in all patients. Recurrence-free survival period (RFS) ranged from 10.2 to 120.0 months, with a mean RFS of 46.8 months. Fifteen percent of the cases recurred before 1.5 years from diagnosis. Among the 20 patients, 10 suffered a single recurrence and 10 suffered more than one recurrence. Thus, the series included 20 primary tumors (PT), 19 first recurrences (RC1), and 11 subsequent recurrences (RC+). The main clinical features are shown in Table 1.

Histologically, all 20 PT demonstrated characteristics of MN. Following the last WHO classification [1], primary MNs were diagnosed as grade 1 in 8 cases, grade 2 in 10 cases, and grade 3 in 2 cases. All the recurrences were diagnosed as grade 2 and 3, in which there were 14 grade 2 and 5 grade 3 recurrences (Table 1). For subsequent analysis, grades 2 and 3 were considered together. Primary grade 1 MNs were diagnosed upon histology as transitional (four cases), meningothelial (three cases), and fibrous (one case). All these grade 1 cases showed less than three morphologic criteria of aggressiveness: High cell densities were found in one case, nuclear atypia was observed in two cases, prominent nucleoli were observed in one case, sheeting was observed in four cases, necrosis was observed in two cases, and mitosis was observed at an average of 1.1 ± 0.5. All grade 2–3 primary MNs presented three or more morphologic criteria of aggressiveness: high cell density was observed in 7 cases, nuclear atypia was observed in 5 cases, prominent nucleoli were observed in 6 cases, sheeting was observed in 10 cases, necrosis was observed in 7 cases, and mitosis count showed an average of 3.5 ± 1.1. The infiltration of the dura was quite frequent, and it is observed in seven grade 2–3, and in five grade 1; but the infiltration of the CNS surrounding structures was only found in one grade 2–3 meningioma. The Ki-67 index showed similar values in grade 1 and grade 2–3 PTs as an average 4.7% of the cells in the former and 4.1% in the latter.

### 3.2. Primary Meningiomas with Similar Outcomes Displayed Genetic Differences Depending on the Grade

**Figure 1:**
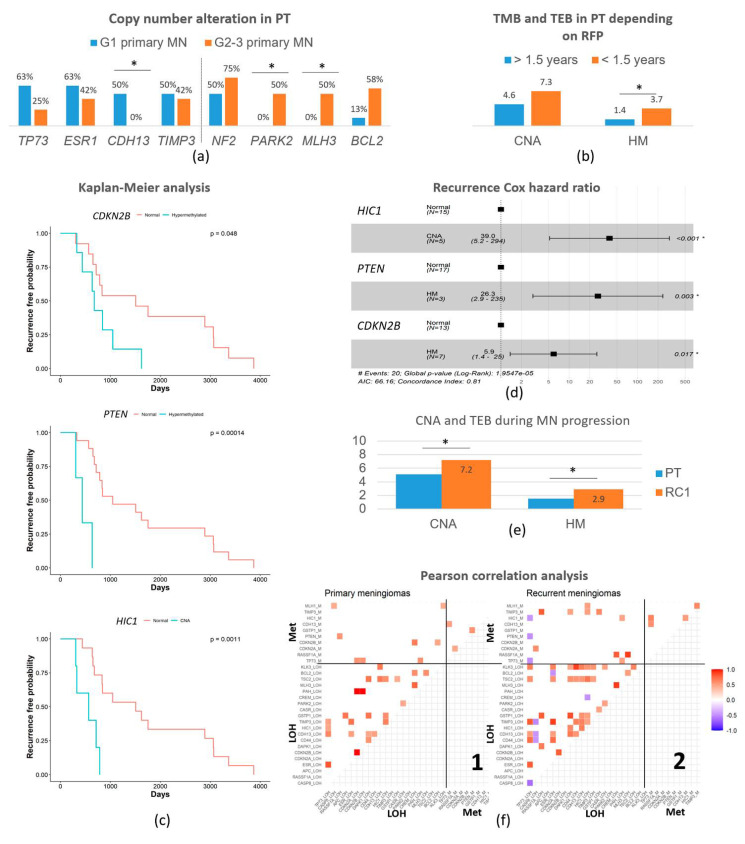
Genetic and epigenetic main characteristics. (**a**) Outstanding somatic copy number alterations on primary meningiomas depending on the tumor grade. (**b**) Copy number alterations and tumor epimutation burden (average number of epigenetic changes) according to recurrence-free survival period, divided into less than 1.5 years to the first recurrence or more than 1.5 years to the first recurrence. (**c**) Kaplan–Meier analysis of recurrence-free survival shows three independent prognostic factors: *CDKN2B* and *PTEN* epigenetic alterations (HM), and *HIC1* copy number alteration (CNA). In each graph, the wild-type situation is in red and HM or CNA is in blue. (**d**) Cox Hazard ratio data to corroborate the strength of Kaplan–Meier analysis. (**e**) Evolution of the genetic and epigenetic burden from primary meningiomas to the first recurrence. (**f**) Pearson’s correlation analysis on primary meningiomas (PT) −1, and their first recurrences (RC1) −2. Positive correlations are in red meaning statistical co-alteration of a pair of genes, both in 1 and in 2. Negative correlations are in blue, meaning statistical alternations in the alteration of a pair of genes (a change detected in 1 is wild-type in 2 and vice-versa). The lower-left quadrant correlates the different CNAs among themselves on PT (1) and RC1 (2). The upper-left quadrant correlates the different CNA versus the different hypermethylation on PT (1) and RC1 (2). The upper right quadrant correlates the different hypermethylation among themselves on PT (1) and RC1 (2). The lower-right quadrant is empty. Abbreviations: GI, grade 1; GII-III, grade 2–3; MN, meningioma; PT, primary tumor; RC, recurrence; CNA, copy number alterations; TEB, tumor epimutation burden. (*) indicates *p* < 0.050.

The average of CNA detected in PT was 4.9 ± 0.6 CNA per case, showing a similar tumor mutation burden (TMB) in the PT of the different WHO grades: It was 5.1 ± 1.1 CNA per case in grade 1 PT and 4.9 ± 0.7 in grade 2–3 PT (Table 2). The *NF2* gene showed a loss of heterozygosity (LOH) in 65.0 % of the PT. It showed LOH in 50% of grade 1 PT, and 75% of grade 2–3 PT (*p* = 0.029). Regarding other analyzed genes, the ones that displayed CNA the most were *TP73* in 1p36 (40%); *ESR1* and *PARK2* in 6q (50 and 35.0%, respectively); *BCL2* in 18q21 (40.0%); and *TIMP3* in 22q12 (45.0%) (Table 2). Comparing MN from different grades, we found that grade 1 PT demonstrated a significantly higher frequency of CNA on *CDH13*, showing losses in 50% of grade 1 PT and none in grade 2–3 PT (*p* = 0.014). Conversely, grade 2–3 PT showed significantly higher rates of alterations in *MLH3* (14q), accounting for 50.0% of grade 2–3 PT and none in the grade 1 PT that recurred (*p* = 0.048), and *BCL2* (18q) in 58.3% of grade 2–3 vs. 12.5% in grade 1 (*p* = 0.054) (Figure 1a).

The hypermethylation study showed a tumor epimutation burden (TEB) measured as the average genes hypermethylated per case of 1.5 ± 0.3. PT showed an average of 1.1 ± 0.5 TEB in grade 1 PT and 2.1 ± 0.3 in grade 2–3 PT (*p* > 0.05). The most frequently hypermethylated genes in PT were *RASSF1A*, *CDKN2A/B*, and *CDH13* (Table 2). No epigenetic difference in an isolated gene reached statistical meaning per se on these PT. However, the increase in Ki67 index in the cases showing CNA of *TP73* (7.7% vs. 2% in wt), *ESR1* (6.7% vs. 1.7%), and CD44 (7.7% vs. 3.5%) is notorious. Interestingly, cases with losses of *NF2* showed an average of 2.2 ± 1.3 hypermethylated genes while it was 0.4 ± 0.5 in the cases with *NF2* wild-type (*p* = 0.001).

### 3.3. Recurrence-Free Survival Associated with the Epigenetic Burden and Specific Changes

We analyzed the association between these genetic (CNA) and epigenetic alterations (HM) and also the recurrence-free survival period (RFS) until the first recurrence. Independently of the tumor grade, patients that recurred before 1.5 years (n = 3) presented 3.7 ± 0.3 HM genes per case while patients that suffered recurrences after 1.5 years (n = 17) presented 1.4 ± 0.3 (*p* = 0.007) (Figure 1b). It was significant that all these cases that recurred early showed the epigenetic alteration of *RASSF1A* (*p* = 0.031) and 2/3 of them in *PTEN* (*p* = 0.046). Regarding CNA burden, the average was also higher but not significant, reaching 7.3 ± 1.2 genes in the early recurrent patients but 4.6 ± 0.7 in the late ones (*p* > 0.05).

Recurrence-free survival (RFS) curves were calculated on primary meningioma samples using the Kaplan–Meier analysis and the differences between curves were assessed by the log-rank test. Interestingly, the CNA of *HIC1* and HM of genes *CDKN2B* and *PTEN* showed statistical associations with the time-to-recurrence curves (Figure 1c). The CNA of *HIC1* caused a 3.2-fold reduction in RFS (*p* = 0.002) and the epigenetic alteration of *PTEN* and *CDKN2B* resulted in a 3.5 and 2.2 times drop in the time to the first recurrence, respectively (*p* = 0.001 for *PTEN* and *p* = 0.021 for *CDKN2B*). The MN with these genetic and epigenetic alterations had a significantly shortened time until recurrence. To confirm these results, we used the Cox proportional-hazards regression method, confirming a higher hazard ratio of recurrence in the same period for tumors with a CNA of *HIC1* (HR = 39, 95% CI between 5.2 and 294, *p* < 0.001), HM of *PTEN* (HR = 26.3, 95% CI between 2.9 and 235, *p* = 0.003), or HM of *CDKN2B* (HR = 5.9, 95% CI between 1.4 and 25, *p* < 0.017) than for tumors without these alterations (Figure 1d).

Afterwards, we explored the status of chromosomes frequently altered in MN 22, 1, and 14 by FISH as a second technique to validate our MLPA results. FISH analysis revealed losses of chromosome 22 in 60.9% of the samples, losses of chromosome 1p in 38.1% of cases, and losses of chromosome 14q in 40.0%. Comparing them to the MLPA data obtained by *NF2* for chromosome 22, *TP73* for 1p, and *MLH3* for 14q (Table 2), we found a concordance of 88.9% when the status was wt, and a concordance of 92.9% for the losses detected for chr. 22. This resulted in a Cohen’s K (chr. 22) = 0.817, which means an almost perfect agreement. For chr. 1, we found a concordance of 84.6% for wt samples and 75.0% for the losses, which led to a Cohen’s K (chr. 1) = 0.512 and means a moderate agreement. Finally, for chr. 14 we found concordance for wt status in 83.3% and for losses in 87.5%, resulting in a Cohen’s K (chr. 14) = 0.694, which means a substantial agreement. Thus, globally, the concordance was good.

### 3.4. Copy Number Alterations and Hypermethylation Increased in the First Recurrence

All cases included in this study recurred; thus, we could analyze the genetic and epigenetic features that changed from the PT to the RC1. The average of CNA increased from the initial 4.9 ± 0.6 up to 7.2 ± 0.7 genes per case in those first recurrences (RC1, *p* = 0.021). Regarding the epigenetic status, the average number of hypermethylated genes was 1.5 ± 0.3 in PT and 2.9 ± 0.6 in RC1 (*p* = 0.029). Globally, the first recurrence (RC1) displayed subtle increases in CNA and HM, affecting a variety of genes, but no one reached statistical significance (Figure 1e). However, it is worthy to mention that *NF2* deletion that was present in 65.0% of PT increased to 78.9% in RC1. Other changes were less frequent but displayed increases of 2.5-fold or more for CNA on *CASP8* (in 2q) and *TSC2* (in 16p), which raised from 10.0% of PT to 26.3% of RC1 in both cases; CNA on *CDH13* (in 16q) from 20.0% to 36.8%; CNA on *KLK3* (in 14q) from 10.0% of PT to 31.6% of RC1; and HM in *GSTP1* (in 11q) from 5.0% of PT to 26.3% of RC1 (Table 2).

Pearson’s correlation analyses offered a correlation matrix between genetic and/or epigenetic alterations in these primary meningiomas and first recurrences that showed numerous significant differences (Figure 1f). PT showed less statistical correlations (25 correlations) that were weaker and only positive between CNA, offering a landscape of co-alterations of those genes in PT (Figure 1(f1)), lower left quadrant) different to RC1. RC1 displayed more statistically significant correlations (46 correlations) with higher positive and negative correlation coefficients; this meant that RC1 displayed both co-alterations and alternative alterations that affected pairs of genes involved in distinct pathways (Figure 1(f2)), lower left quadrant). The CNA of *CDH13* is the alteration correlating with CNA in most other genes in both PT (6 correlations) and RC1 (10 correlations). Most correlations between CNA events were conserved in RC1 vs. PT despite the increase and occurrence of those exclusive changes.

Interestingly, the CNA in *CASP8* did not show any association with other CNA in PT but showed negative correlations with CNA in four genes in RC1 (*CD44*, *CDH13*, *TIMP3*, and *TP73*). *KLK3* also showed only one correlation for CNA in PT, whereas it correlates with nine other CNA in RC1. Associations between CNA and epigenetic changes showed 8 positive correlations in PT (Figure 1(f1)), upper left quadrant) while it showed 10 positive correlations and 3 negative correlations in RC1 (Figure 1(f2)), upper left quadrant). None of these correlations can be explained by the proximity of gene chromosomal *loci*. These last correlations were in general not conserved between primary and recurrent meningiomas as most of the correlations found between CNA and hypermethylation in RC1 were not observed in PT (only 1 out of 14). Conversely, only 1 out of the 10 correlations between CNA and hypermethylation in PT was present in RC1. Noteworthy, *TP73*, a well-known tumor suppressor gene reported previously in meningiomas, showed no correlation in PT but demonstrated three negative correlations in RC1 with DNA hypermethylation (*TP73* itself, *PTEN*, and *HIC1*). Finally, correlations between hypermethylation events (upper right quadrants) were scarce both in PT and RC1 (only 3 and 4, respectively). The lower right quadrants are empty by the method.

### 3.5. Recurrence Genetic Background Depended on the Histologic Grade of the Primary Tumor

**Figure 2:**
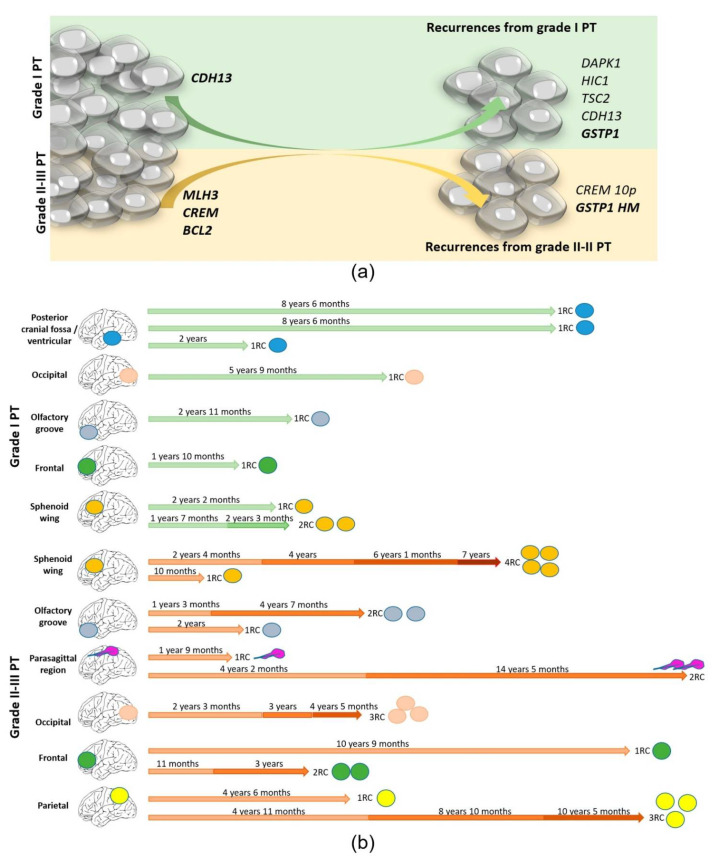
(**a**) The genetic and epigenetic changes in MN are different between grade 1 PTs and higher grades but frequent in both groups. Grade 2–3 PTs offer consistent alterations located in a few chromosomes (1, 14, and 18), while grade 1 PTs display randomly distributed alterations. RCs from primary grade 2 to 3 MN introduce a few changes because it is higher than its initial burden, while the progression of grade 1 MN, which displayed more random alterations initially, introduced many concrete changes when it recurs (**b**). Timing of the disease according to the grade of the PT (grade 1 in upper part/ grade 2–3 in lower part) indicating the location of the primary in each case.

The lapse of time from the PT to the first recurrence was 53.5 ± 13.8 months for recurrent grade 1 PT and 45.0 ± 11.3 for grade 2–3 PT (*p* > 0.050), but their genetic and epigenetic changes were different as is described above. Thus, we analyzed the genetic background of the first recurrence of these cases (n = 19) according to the WHO grade of the primary tumor from which each one evolved instead of their own WHO grade (Figure 2). As an average, we found 7.5 ± 1.4 CNA per case in RC1 from grade 1 PT (compared to 4.9 on grade 1 PT, *p* > 0.050) and 6.9 ± 0.8 in RC1 from grade 2 to 3 (compared to 4.8 on grade 2–3 PT, *p* > 0.050). Regarding epigenetics, we found 2.9 ± 0.8 HM genes per case in RC1 from grade 1 PT (compared to 1.1 on grade 1 PT, *p* > 0.050) and 3.0 ± 0.8 in RC1 from grade 2 to 3 PT (compared to 1.8 on grade 2–3 PT, *p* > 0.050). Thus, globally, the figures seemed similar. Although the burden was similar, it is of interest that we found that RC1 from grade 1 PT displayed CNA in *DAPK1* with a significantly higher frequency, showing losses in 50% of RC1 from grade 1 PT and none in RC1 from grade 2 to 3 PT (*p* = 0.018). A similar observation was observed for *CDH13*, which was altered in 62.5% of RC1 from grade 1 PT and in 18.2% of RC1 from grade 2 to 3 PT (*p* = 0.048); for *HIC1*, it was altered in 75.0% of RC1 from grade 1 PT and in 20.0% of RC1 from grade 2 to 3 PT (*p* = 0.031); for *GSTP1*, it was altered in 8% of RC1 from grade 1 PT and none in RC1 from grade 2 to 3 PT (*p* = 0.058). The CNA in *TSC2* did not reach a significant meaning but it was found in 50.0% of RC1 from grade 1 PT and in 9% of RC1 from grade 2 to 3 PT (*p* = 0.071). The opposite happened in *CREM*, which displayed CNA in 63.6% of RC1 from grade 2 to 3 PT and in 12.5% of RC1 from grade 1 PT (*p* = 0.037) and also with the methylation status of *GSTP1*, which was hypermethylated in 45.5% of RC1 from grade 2 to 3 PT and none in RC1 from grade 1 PT (*p* = 0.040).

### 3.6. Genetic Evolution in Subsequent Relapses Was Heterogeneous

Out of the 20 patients studied, 13 suffered only one recurrence but 7 suffered additional recurrences. As expected, 87.5% of multiple recurrences came from grade 2 to 3 primary meningiomas and only 12.5% came from grade 1 primary MN (*p* = 0.040). The timing according to the grade of the PT and the location is represented in Figure 2b. The genetic burden on these RCs continued to increase up to 7.5 ± 0.6 CNA per case (*p* = 0.003), and all of them displayed more than five CNAs among the *loci* explored. Similar findings were detected regarding TEB, with an increase of up to 2.9 ± 0.4 HM genes per case in RCS (*p* = 0.015). The recurrence-free survival period until the first recurrence was significantly shorter in patients that suffered multiple recurrences (27.92 ± 4.8 months) than in patients that suffered only one MN RC (68.7 ± 13.9 months, *p* = 0.018). Heterogeneous alterations were found in the different cases showing a random genetic and epigenetic evolution. It is noteworthy that homozygous deletions that were scarce in RC1 occurred from RC2 and were sustained in subsequent RCs. The most outstanding findings were the homozygous deletions in *TP73* in 4/7 RC2 and hypermethylated in the rest. CNAs were found in *CD27*, *CDKN2A/2B*, and in *CDH13*, in two RC2, and the hypermethylation of *GSTP1* was observed in 3/7 RC2. Other alterations seemed to be more randomly distributed, affecting *VHL*, *FHIT*, *KLK3*, or *DAPK1.*

## 4. Discussion

Recurrent MNs are lurid causes of disability and their frequencies, together with the evolution in the conception of wellness and aging, have changed the status quo: middle-aged people that develop this intracranial neoplasm suffer many complications derived from the disease. Their high frequency, delicate location, complicated risk prediction, the presence of different morphological patterns within the same sample, and interobserver biases [1] underline the need for additional objective molecular markers to improve the clinical management of affected patients.

The series presented here sheds light on the clinical, genetic, and epigenetic features of matched samples of primary and recurrent tumors from the same patients. It constitutes, to our knowledge, the second-largest series of these characteristics: 50 samples that represent the primary tumor of 20 patients, half of them suffering one recurrence and the other half suffering more than one recurrence [19]. We deepen on the stepwise progression of the disease by analyzing features on those recurrences. Studying this type of series offers some results different from the standard descriptions of MN as a consequence of the selected cohort. The first one is that our patients, who suffered recurrences in all the cases, are younger than the average in the literature [1], and they are more than 2/3 under 60 years old at diagnosis. The second differential finding is the inversion of the ratio of men:women (here set at 1.5:1), which agrees with previous descriptions about aggressive MN in men and is also concordant with the highest frequent location on the skull’s base [20]. Whether surgical difficulties that lead to Simpson’s grade 2 or higher resections are responsible for the recurrence [21,22] or whether they occur because of the selective pressures in that region [20,23,24] continue to be unanswered, but in the present study, all resections are macroscopically complete, suggesting an important effect by this particular region. Regarding histological features, sheeting was the main histopathological characteristic in this series of tumors indistinctively of the tumor grade.

Here, we show that genetic and epigenetic changes are abundant in these aggressive MN, similarly to what is usually described for high-grade MN. We also agree that these DNA changes increase with the grade of the tumor and with the development of recurrences in concordance with previous reports [1,4,25]. It has been described that radiosurgery and/or radiotherapy increase tumor instability in addition to cause other side effects [7,21,25]. Our patients did not receive any adjuvant therapy before the first surgery. However, all cases with multiple recurrences progressed to grade 2 tumors at some moment of the disease, and when the gross total resection of a recurrence was not possible, post-surgical fractioned radiotherapy was administrated. Although this could influence the continuous increase in tumor instabilities detected, it does not detract from the changes detected between PT and RC1 that were different depending on the grade of primary (there was only one grade 3 PT with adjuvant therapy before RC1). In addition, the different growth rates between genetic and epigenetic burdens found are also notable. The most common alteration, in agreement with the literature, is the loss of *NF2* in chromosome 22q [1,8,10,26]. It is an early event occurring in half of the primary tumors analyzed, but its loss increases with tumor grade and with the progression. Indeed, we observed this loss in the last recurrence of every of the cases that developed multiple recurrences. Interestingly, primary *NF2*-altered MNs show higher TEB in this series, and TEB is statistically associated with early recurrences in concordance with previous descriptions [7,12,27,28]. The involvement of both types of changes in aggressiveness raises the need for improving the comprehension of common underlying mechanisms between them. On the other hand, CNAs are high from the debut of the disease and increase only subtly.

Regarding CNAs, the main affected genes in this series of primary MN that recurred are located on chromosomes 22q (*TIMP3*), 1p (*TP73*), 6q (*ESR1* and *PARK2*), and 18q (*BCL2*); the association among these chromosomes’ alterations and tumor recurrence has been previously demonstrated [1,4,29]. The increase in CNAs in primary grade 2–3 MN affecting 14q (*MLH3*), 18q (*BCL2*), and 10p (*CREM*) is significant. Barresi et al. demonstrated the association of 18q losses with RFS in atypical meningiomas [26], in agreement with a shrinkage of 17 months in our series. However, our data did not reach statistical significance. When all these primary MNs progress, the highest rates in their RC1 affect *TIMP3*, *TP73*, *ESR1*, and *PARK2* again. These findings are consistent with cytogenetic descriptions of losses on those chromosomes in MN and the development of recurrences [1,4,29]. From them, losses on *TIMP3* (22q) have been suggested to take part in reduced apoptosis [30,31] or angiogenesis [32], and our finding supports the extensive belief that additional genes located on 22q should contribute to MN tumorigenesis [3,4]. The influence of estrogens in MN has been widely discussed on MN, with many discrepancies in the literature but little has been described at the genetic level [33,34]. Both *ESR1* and *PARK2* are located in a frequently altered chromosomal region in MN (6q), and both display effects over the Wtn/b-catenin pathway [35,36,37,38,39]. Interestingly, it has been suggested that this pathway influences the formation of recurrences in MN [38,39], emphasizing the interest in further research on the effects of *ESR1* or *PARK2* losses. Regarding *CREM*, it is located in chromosome 18, and its loss is widely associated with high-grade MN and progression [1,40,41]. *BCL2* immunostaining has been reported to increase with tumor grades in MN [21], but the dysregulation of the control of apoptosis could impact in both directions [42], suggesting a potential role of *BCL2* losses in cell-death control that would agree with our findings. Finally, *MLH3* losses on 14q completes the set of altered genes that are located in cytogenetic hotspots of meningioma [1,29]. The co-alteration of these genes could be a mechanism for acquiring aggressiveness in some subgroups of meningiomas. Although CNAs seem to be high since the diagnosis in these potentially recurrent PTs, it increases significantly during the disease, as we can corroborate with the analysis of the different recurrences. This fact is not only aligned with the idea that tumor instability increases with MN progression [26] but also highlights the high rate of random CNAs in grade 1 primary meningiomas that behave aggressively.

Our most outstanding finding, thanks to the comparison of paired PT-RC, is that TEB has a negative impact on recurrence-free survival and it also increases during the progression of the disease, similarly to previous descriptions from ours and others [12,43,44]. Although our previous work explored the epigenetic burden effect as a ‘yes’ or ‘no’ issue, here, we delve deeper into its ability to shorten the disease-free survival period. Our results emphasize an important role of the epigenetic inactivation of *CDKN2B* and *PTEN* in the timing of the disease. Both genes, together with CNA in *HIC1*, demonstrate independent prognostic value. Although losses in *PTEN* (10p) are related to the progression of this disease [1,40,41], no references to its epigenetic inactivation have been reported. Of note is that, in contrast to the abundant literature about the influence of *CDKN2B* and *PTEN* on MN, as far as we have reviewed the literature, there is no previous study describing *HIC1* in MN. However, its epigenetic inactivation has been described in other benign brain tumors such as intracranial ependymoma and medulloblastoma [45,46]. Unexpected Pearson’s correlations suggest that different pathways activated in tumor initiation compared to progression: CNAs in *HIC1* were inversely associated with HM in *PTEN* on PT and in itself, offering at least two clusters of genes differentially affected when the tumor progresses and both causing a shortened recurrence-free survival. This novel finding highlights the heterogeneity of MN and plays in favor of the frequently proposed model of clonal evolution in meningioma [47,48,49,50].

Our next question was whether or not recurrences from grade 1 PT that progress are genetically/epigenetically similar to those from grade 2 to 3 PT. With a marked involvement of the aforementioned chromosomes 14, 6, and 10, we found clusters of alterations of interest in the recurrences that depend on the grade of the primary tumor. The high rate of genetic/epigenetic changes that are present on grade 2–3 tumors from the beginning of the disease softens its increase during progression, while the substantial boost on alterations becomes most evident in the recurrences that come from grade 1 PT. Interestingly, *GSTP1* seems to represent a bridge on these recurrences as it is the only individual gene for which its inactivation was significant in RC from grade 2 to 3 tumors via epigenetic alteration and from grade 1 tumors via copy number alteration. *GSTP1* (11p) hypermethylation has been previously reported in MN literature [28,43], But it is not associated with an additional parameter. Different studies report that *GSTP1* silencing activates JNK and ERK1/2 pathways to explore apoptosis and uncontrolled growth [51,52,53,54]. In either cases, the adjacent connection to cell-death programs lead us to think of a major role for the apoptosis–necroptosis–autophagy axis. A subtle association between losses on *BCL2* and *PARK2* is of interest, which would promote autophagy in MN. On the other side, the lack of an influence of *TSC2* over the mTOR pathway would result in reduced autophagy that could be reinforced by the reduction in *DAPK1* phosphorylating Beclin [55,56]. These interrelations are more notorious in the multiple RCs that we have had the chance to evaluate in this series.

Patients that developed more than one RC offer a wide spectrum of alterations. DNA methylation profiling seems to be the most robust method for estimating the risk of recurrence [26], although it can be unaffordable for many clinical facilities. Our data emphasize not only the interest of studying the accumulation of epigenetic changes in MN progression but also the possibility of its assessment by MLPA. It is a cost-effective technique available for almost every kind of laboratory that in addition is of straightforward interpretation. Of the entire epigenetic burden found in MN progression, the most outstanding is *GSTP1* hypermethylation occurring in five of seven RC2 that presented simultaneously *TP73* loss. This may represent a pathway different than others that affected *CASP8-CASR-PARK2-MLH3* from the first recurrence. Surprisingly, both recurrences that displayed hypermethyltion in *GSTP1* or the ones with other changes affecting the *MLH3-CREM-BCL2* axis, *DAPK1-HIC1-TSC2* axis, or even displaying stochastic alterations of *VHL* and/or *FHIT* and/or *KLK3* and/or *DAPK1* underline the involvement of apoptosis-autophagy in MN aggressiveness. Although previous descriptions associating these genes are scarce, their adjacent relation with cell-death pathways is remarkable. In addition to the above-described functions, *VHL* acts as a target recruitment subunit in the E3 ubiquitin ligase complex (as *PARK2*), which recruits hydroxylated hypoxia-inducible factor (HIF) and its loss may help apoptosis evasion. *FHIT* plays a role in the induction of apoptosis via *SRC* and *AKT1* signaling pathways; *DAPK1* is involved in apoptosis and autophagy; *KLK3* is an androgen receptor responsive gene and its downregulation has been associated with increased autophagy [39]. These findings, taken together, underline the unexplored role of apoptosis autophagy as a relevant mechanism of MN progression worthy of further research.

## 5. Conclusions

The study of tumors’ self-evolution is important for understanding disease progression and developing novel therapies. Most previous studies focused on which meningiomas recurred and which did not within the histological grades classified by the WHO, but to the best of our knowledge, this is the second-largest pair-matched collection and the first report studying the recurrence by considering the grade of the primary neoplasm with an emphasis in grade 1 MN with aggressive behavior. We observed similarities not only between these recurrent tumors but we also identified differences in the prognosis thanks to the wide follow-up conducted. The different statistical analysis shows interesting interrelations between apoptotic genes and autophagy, and a surprising involvement of *GSTP1*, *BCL2 DAPK1*, and *HIC1* that deserves further consideration in meningioma. Our approach sheds some light on MN heterogeneity and emphasizes the importance of introducing user-friendly and cost-effective methods in the molecular characterization of brain tumors in the daily clinical routine.

## Figures and Tables

**Table 1:** Clinical, histopathological, and genetic summary.

Case	Sample	Grade	Age	Sex	Location	Tumor Size (cm)	RFS (Months)	Histology	Mitosis	CNA	HM
1	PT	1	64	W	Olfactory groove	6.5	-	Transitional	1	0	3
	RC1	2	67			5.0	34.9	Atypical	3	2	4
2	PT	1	34	M	Convexity (frontal)	8.0	-	Meningothelial	2	3	0
	RC1	2	36			7.5	21.8	Atypical	2	2	2
3	PT	1	57	W	Posterior cranial Fossa	4.0	-	Meningothelial	2	4	0
	RC1	2	59			4.0	23.9	Atypical	10	9	6
4	PT	1	52	W	Posterior cranial Fossa	4.0	-	Meningothelial	3	3	4
	RC1	2	60			1.5	102.3	Atypical	5	8	6
5	PT	1	7	M	Ventricular	3.0	-	Fibrous	2	5	0
	RC1	2	16			3.7	102.1	Atypical	5	9	0
6	PT	1	66	W	Convexity (occipital)	4.0	-	Transitional	3	9	1
	RC1	2	74			3.3	69.4	Atypical	5	12	3
7	PT	1	53	M	Sphenoid wing ring	7.0	-	Transitional	3	7	0
	RC1	2	58			5.0	26.3	Atypical	3	6	0
8	PT	1	58	M	Sphenoid wing ring	2.0	-	Transitional	4	10	1
	RC1	2	59			6.0	18.9	Atypical	5	12	2
	RC2	2	60			1.1	27.7	Atypical	5	13	3
9	PT	2	60	M	Sphenoid wing	na	-	Atypical	3	5	4
	RC1	2	61			na	10.2	Atypical	4	6	2
10	PT	2	48	W	Na	0.0	-	Atypical	6	7	2
	RC1	2	57			0.0	112.3	Atypical	6	12	7
11	PT	2	68	W	Convexity (parietal)	7.0	-	Atypical	2	5	1
	RC1	3	72			3.0	54.1	Anaplastic	11	7	9
12	PT	2	49	W	Convexity (frontal)	4.0	-	Atypical	2	0	1
	RC1	3	59			5.0	129.0	Anaplastic	10	9	2
13	PT	2	65	M	Parasagittal region	6.5	-	Atypical	3	3	3
	RC1	3	67			4.0	21.3	Anaplastic	3	3	3
14	PT	3	46	M	Olfactory groove	5.0	-	Anaplastic	5	4	1
	RC1	3	47			3.5	22.6	Anaplastic	5	4	3
15	PT	3	59	M	Convexity (frontal)	6.0	-	Anaplastic	10	8	3
	RC1	3	60			na	11.0	na	na	na	na
	RC2	3	62			3.5	36.0	Anaplastic	7	7	1
16	PT	2	22	W	Parasagittal region	5.0	-	Atypical	3	3	2
	RC1	2	26			0.0	50.2	Atypical	6	7	2
	RC2	2	36			7.0	173.0	Atypical	4	10	3
17	PT	2	59	M	Olfactory groove	3.5	-	Atypical	10	9	4
	RC1	2	61			5.0	14.7	Atypical	4	11	3
	RC2	3	64			8.0	55.2	Anaplastic	12	14	4
18	PT	2	66	M	Convexity (parietal)	5.0	-	Atypical	2	5	1
	RC1	2	71			4.0	58.5	Atypical	2	5	1
	RC2	2	74			5.0	106.2	Atypical	5	6	4
	RC3	2	76			5.0	125.3	Atypical	4	8	4
19	PT	2	53	M	Sphenoid wing ring	7.0	-	Atypical	4	6	1
	RC1	2	56			7.0	28.1	Atypical	4	6	1
	RC2	2	57			5.0	47.8	Clear-cell	4	8	1
	RC3	2	60			4.0	72.8	Clear-cell	6	7	2
	RC4	3	61			0.0	83.1	Clear-cell	5	5	2
20	PT	2	53	M	Convexity (occipital)	7.0	-	Atypical	3	4	3
	RC1	2	56			4.3	27.8	Atypical	2	5	1
	RC2	3	56			3.3	39.1	Anaplastic	5	5	1
	RC3	3	57			3.0	52.6	Anaplastic	10	8	1

Abbreviations: HM, number of genes hypermethylated; M, man; na, no available; CNA, somatic copy number alterations; W, woman.

**Table 2:** Distribution of genetic and epigenetic alterations.

			Global		PT		G1 PT		G2–3PT			RC1	
				n = 50		n = 20		n = 8		n = 12			n = 19
	**Gene**	Locus		CNA		**4.9 ± 0.6**		5.1 ± 1.1		4.9 ± 0.7			7.2 ± 0.7 *
Loss of heterozygosity	***TP73***	01p36	27	54.0%	8	40.0%	5	62.5%	3	25.0%		10	52.6%
	***CASP8***	02q33-q34	9	18.0%	2	10.0%	1	12.5%	1	10.2%		6	31.6%
	***FHIT***	03p14.2	4	8.0%	1	5.0%	1	12.5%	0	0%		2	10.5%
	***RASSF1A***	03p21.3	4	8.0%	1	5.0%	0	0%	1	8.3%		3	15.8%
	***VHL***	03p26-25	2	4.0%	0	0.0%	0	0%	0	0%		2	10.5%
	***CASR***	03q13.3-q21.1	12	24.0%	4	15.0%	2	25%	2	16.7%		5	26.3%
	***APC***	05q21	4	8.0%	0	0.0%	0	0%	0	0		1	5.3%
	***ESR1***	06q25.1	29	58.0%	10	50.0%	5	62.5%	5	41.7%		10	52.6%
	***PARK2***	06q26	19	38.0%	6	30.0%	0	**0%**	**6**	**50.0%**	*	8	42.1%
	***CDKN2A***	09p21	6	12.0%	1	5.0%	0	0	1	8.3%		3	15.8%
	***CDKN2B***	09p21	5	10.0%	1	5.0%	0	0	1	8.3%		2	10.5%
	***DAPK1***	09q34.1	11	22.0 %	2	10.0%	2	25.0%	0	0%		5	26.3%
	***CREM***	10p11.21	27	54.0%	7	35.0%	2	25.0%	7	58.3%		8	42.1%
	***PTEN***	10q23.31	5	10.0%	0	0.0%	0	0%	0	0%		1	5.3%
	***CD44***	11p13	14	28.0%	3	15.0%	2	25.0%	1	8.3%		7	36.8%
	***GSTP1***	11q13	6	12.0%	2	10.0%	2	25.0%	0	0%		3	15.8%
	***CD27***	12p13.31	6	12.0%	1	5.0%	0	0%	1	8.3%		1	5.3%
	***PAH***	12q23.2	11	22.0%	2	10.0%	0	0%	2	16.7%		5	26.3%
	***MLH3***	14q24.3	19	38.0%	6	30.0%	0	**0%**	**6**	**50.0%**	*	7	36.8%
	***TSC2***	16p13.3	10	20.0%	2	10.0%	2	25.0%	0	0%		6	31.6%
	***CDH13***	16q24	13	26.0%	4	20.0%	**4**	**50.0%**	0	0%	*	7	36.8%
	***HIC1***	17p13.3	19	38.0%	5	25.0%	3	37.5%	2	16.7%		9	42.1%
	***BCL2***	18q21.33	22	44.0%	8	40.0%	**1**	**12.5%**	**7**	**58.3%**	*	9	42.1%
	***KLK3***	19q13.3	11	22.0%	2	10.0%	2	25.0%	0	0%		6	31.6%
	***TIMP3***	22q12.3	29	58.0%	9	45.0%	4	50.0%	5	41.7%		12	63.1%
	***NF2***	22q12.2	39	78.0%	13	65.0%	4	50.0%	9	75.0%		15	78.9%
				HM		**1.5 ± 0.3**		1.1 ± 0.5		2.1 ± 0.3			2.9 ± 0.6 *
Hypermethylation	***TP73***	01p36	11	22.0%	3	15.0%	2	25.0%	1	8.3%		6	31.6%
	***RASSF1A***	03p21.3	23	46.0%	8	40.0%	2	25.0%	6	50.0%		9	42.1%
	***MLH1***	03p21.3	7	14.0%	3	15.0%	1	12.5%	2	16.7%		4	21.1%
	***RARB***	03p24	4	8.0%	2	0.0%	1	12.5%	1	8.3%		2	10.5%
	***ESR1***	06q25.1	4	8.0%	0	0.0%	0	0	0	0		3	15.5%
	***CDKN2A***	09p21	7	10.0%	3	15.0%	0	0%	3	25.0%		3	15.5%
	***CDKN2B***	09p21	19	38.0%	7	35.0%	1	12.5%	6	50.0%		6	31.6%
	***DAPK1***	09q34.1	2	4.0%	0	0.0%	0	0	0	0%		2	10.5%
	***PTEN***	10q23.31	11	22.0%	3	15.0%	0	0%	3	25.0%		7	36.8%
	***GSTP1***	11q13	12	24.0%	1	5.0%	0	0.0%	1	8.3%		5	26.3%
	***CDH13***	16q24.2	16	32.0%	5	25.0%	2	25.0%	3	25.0%		8	42.1%
	***HIC1***	17p13.3	3	6.0%	0	0.0%	0	0	0	0		3	15.8%
	***TIMP3***	22q12.3	4	8.0%	1	5.0%	0	0%	1	8.3%		3	15.8%

PT, primary tumor; RC1, first recurrence, *p*-values are indicated as * when ≤ 0.050.

## Data Availability

All data needed to evaluate the conclusions in the paper are present in the paper. Additional data related to this paper may be requested from the corresponding authors.
